# Dynamics of Immune Responses during Experimental *Mycobacterium kansasii* Infection of Cynomolgus Monkeys *(Macaca fascicularis)*

**DOI:** 10.1155/2018/8354902

**Published:** 2018-06-05

**Authors:** Fangui Min, Lifang He, Yinzhu Luo, Shuwu Huang, Jinchun Pan, Jing Wang, Ruike Wu, Lan Zhang, Meili Chen

**Affiliations:** ^1^Guangdong Provincial Key Laboratory of Laboratory Animals, Guangdong Laboratory Animals Monitoring Institute, Guangzhou 510663, China; ^2^Guangdong Provincial Key Laboratory of Malignant Tumor Epigenetics and Gene Regulation Department of Obstetrics and Gynecology, Sun Yat-sen Memorial Hospital, Sun Yat-sen University, Guangzhou 510120, China

## Abstract

To profile the dynamic changes of immune responses for *M. kansasii* infection, 3 cynomolgus monkeys were experimentally infected with *M. kansasii* by intratracheal inhalation of 1 × 10^6^ CFU bacteria per monkey. Every 2 to 4 weeks, tuberculin skin testings (TSTs) were performed and blood samples were collected for immunoassay. Multiple cytokines in a single sample were measured by Luminex xMAP technologies. IgM and IgA were detected by double-antibody sandwich ELISA. IgG against PPD and 11 *M. tuberculosis* proteins were detected by using of indirect ELISA. At week 16, all animals were euthanized for necropsy and histological analysis. Positivities of TSTs emerged from week 2 to 6 postinfection. Leukocyte counts and T lymphocyte subsets experienced moderate increases. Among 44 kinds of cytokines, 36 kinds of them showed increases of different dynamic types and 8 kinds of them showed no specific changes. Total IgM and IgA showed a transient increase at an early infection stage. Positivities of *M. tuberculosis* specific IgM and IgA emerged as early as week 2 postinfection. All animals showed positive IgG against PPD and negative IgG responses to 38 kDa, MPT64L, TB16.3, 16 kDa, U1, and MTB81 antigens during the infection period. IgG against ESAT-6, CFP10, CFP10-ESAT-6, Ag85b, and 14 kDa antigens reached positive levels. The IgG avidities of PPD, ESAT-6, CFP10-ESAT-6, and Ag85b were all above 50 percent. In conclusion, the data indicate that *M. kansasii* infection in monkeys can induce positivities of TSTs, increases of multiple cytokines, and cross-reactive antibody responses to *M. tuberculosis* antigens.

## 1. Introduction

Mycobacterial diseases are of primary health concern in laboratory nonhuman primates (NHP) and have been constantly monitored and screened in captive NHP colonies. Among mycobacterial diseases, tuberculosis is regarded as the most serious risk to nonhuman primate colonies and human handlers that was mainly caused by *Mycobacterium tuberculosis* complex (MTBC) [1–3]. Nontuberculous mycobacterial (NTM), also called “atypical” mycobacteria, demonstrated a greater incidence of natural infections than that of MTBC [4–6]. In Chinese national standard (GB14922.2-2011) for laboratory NHP, NTM need not to be excluded. Then some Chinese-origin laboratory monkeys used for biomedical research or for export may be infected with NTM. Shipley et al. reported that 6 Chinese-origin rhesus macaques in the absence of disease were confirmed to be infected with *Mycobacterium kansasii* [7].

Though many new approaches have been made in tuberculosis diagnosis, the mainstay among the screening methods used for NHP tuberculosis screening remains the tuberculin skin testing (TST), which could not distinguish MTBC infections from NTM infections for cross-reactivity to mammalian old tuberculin or purified protein derivative [8, 9]. NTM reacting positive TST reaction to mammalian old tuberculin has been repeatedly reported in Old World primates or New World primates since the 1980s. However, it remains unknown whether all NTM infections in NHP may cause positive TST reactions. And the immune responses to NTM infection in NHP were also few reported.

For NTM infection in NHP, *M.* kansasii was one of the most often detectable species [7, 10–12]. As we know, there has been no report on experimental infection of *M. kansasii* in NHP. Here, we describe the clinical workup performed to profile the dynamics of immune responses in cynomolgus monkeys *(Macaca fascicularis)* experimentally infected with *M. kansasii*. A description of leukocyte, T lymphocyte subsets, multiplex cytokine assays, and cross-reactive antibody responses to *M. tuberculosis* antigens is included.

## 2. Materials and Methods

### 2.1. Animals

Three male cynomolgus monkeys of 3~4 years of age were obtained from Guangdong Blooming-Spring Biological Technology Development Co. Ltd. (license number SCXK (Yue) 2014-0027). Their IDs were CM1936, CM1937, and CM1938. All monkeys were routinely tested negative for monkey B virus, simian immunodeficiency virus (SIV), and simian T-cell leukemia virus 1 (STLV-1) by ELISA and simian retrovirus (SRV) by immunofluorescence. After arrival at the biosafety level 2 facility, the monkeys were quarantined for 1 month to exclude the tuberculosis by biweekly TSTs.

### 2.2. Bacterial Infection

*M. kansasii* type strain ATCC 12478 was used for infection. After culture for 3 weeks, bacteria were harvested from Lowenstein-Jenden medium and suspended in PBS. Anesthetized animals were challenged with bacterial suspension by intratracheal inhalation as previously reported [13]. The challenge inoculations were 1 × 10^6^ CFU per monkey. Approximately 16 weeks after inoculation, all monkeys were euthanized by intravenous injection of overdose of ketamine combined with xylazine and necropsied by a pathologist.

Animal use protocols were reviewed and approved by the IACUC of Guangdong Laboratory Animal Monitoring Institute (AAALAC accredited) with a number GDLAMI-IACUC2017005.

### 2.3. Clinical Assessment

Monkeys were observed daily for alterations in behavior, appetite, and coughing. Body weights were recorded biweekly. Purified protein derivative- (PPD-) based TST was performed every 2 to 4 weeks.

### 2.4. Blood Collection

Every 2 to 4 weeks up to 16 weeks, blood samples were obtained via femoral venipuncture from 3 monkeys for clinical hematology, erythrocyte sedimentation rate (ESR), flow cytometry analysis, and sera for immunology by centrifugation.

### 2.5. Hematological Analyses

K_2_EDTA-anticoagulant blood (0.5 mL) was used for hematological automated analyses within 4 hours of sample collection, which were done with an automatic hematology analyser (Sysmex XT-2000iV). 1 mL K_2_EDTA-anticoagulant blood was used for flow cytometry analysis. ESR was measured monthly by using Westergren tubes.

### 2.6. Cytokine Measurement in Sera

To determine as many cytokines as possible by using limited sera, multiplexed immunoassay panels (Luminex xMAP technologies) containing 44 cytokine assays were developed by R&D Systems. Information of selected cytokines was listed in Table 1. Generally, selected cytokines were divided into 3 panels according to the rules that analytes with overlapping bead regions or other incompatibilities would be placed in their own assays. Panel 1 included 6 cytokines, which were TNF RII, IL19, CCL5/RANTES, MIF, MPO, and Galection-3bp. For IL-23 could not be multiplexed with CCL5/RANTES, ICAM-1, IL-12/23 p40, or NCAM-1, panel 2 was prepared only for IL23. The other 37 cytokines were equipped in the panel 3. The testings were performed according to the manufacturer's protocol.

### 2.7. Antibody Detection

Serum concentrations of total IgM and IgA were measured by double-antibody sandwich ELISA kits (Ref: GN-M61132, Lot: 201804; Ref: GN-M61115, Lot: 201804). OD_450_ values of *M. tuberculosis* specific IgM and IgA (TB-IgM and IgA) were obtained by using of double-antibody sandwich ELISA kits (Ref: GN-M61150, Lot: 201804; Ref: GN-M61162, Lot: 201804). All operating procedures were performed according to the manufacturer's protocol.

IgG were analyzed by ELISA as described [14]. PPD and 11 kinds of recombinant *M. tuberculosis* proteins (Ag85b, MPT64L, U1, 16 kDa, TB16.3, 38 kDa, 14 kDa, CFP10, ESAT-6, CFP10-ESAT-6, and MTB81) purified to homogeneity from *Escherichia coli* were used as coating antigens for indirect ELISA. Besides MTB81, the other 10 recombinant *M. tuberculosis* proteins had been used for IgG detection in our previous report [14, 15]. MTB81 was also an antigen with high potential for human *M. tuberculosis* diagnosis but lack of usage in nonhuman primate [16].

### 2.8. IgG Avidity

Avidities of IgG against PPD, ESAT-6, CFP10-ESAT-6, and Ag85b were analyzed as previously reported with modifications [17, 18]. Briefly, each serum sample was added and incubated in 2 wells of plates coated with every antigen. After washing the plates incubating with diluted serum samples, 2 wells were added with 6 M urea in PBS and PBS, respectively, and incubated for 10 min. Then, the plates were washed and performed as normal procedures.

The avidity indices (AIs) were calculated by comparing the absorbances of the untreated samples and treated samples as following: AI (%) = (absorbance treated sample/absorbance untreated sample) × 100. According to the measurement of specific IgG avidity for viral infection, IgG with AIs higher than 50% were considered to be high-avidity antibodies. AIs of low-avidity antibodies were lower than 30% [17, 18].

### 2.9. Data Analysis

All the statistical procedures were performed by GraphPad Prism Software Inc. (San Diego, CA, USA). Between-group differences were analyzed by Student's *t*-test.

## 3. Results

### 3.1. Animal Infection Status

The infectious outcomes were listed in Table 2. During the infection period, all monkeys showed no obvious clinical symptoms, including their mental status, body weight, conditions of diet, urine and excrement, and activities of limbs. At necropsies, monkey 1938 presented consolidation in the left middle lobe of the lung which was proved to be interstitial pneumonia with noncaseous granulomas by microscopic examination. Monkeys 1936 and 1937 showed no gross or histopathological lesions. None but the lung of monkey CM1938 was detected with bacteria of 5000 CFU/g in the left middle lobe.

### 3.2. Total Leukocyte and Leukocyte Populations

**Figure 1:**
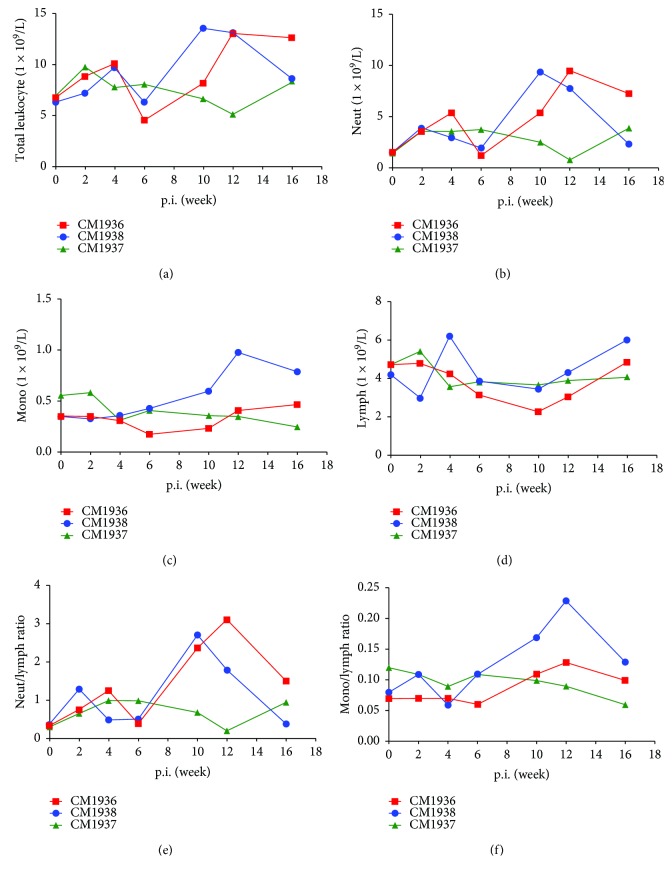
Dynamic changes of total leukocyte and leukocyte populations. (a) Number of total leukocyte. (b) Number of neutrophils. (c) Number of monocytes. (d) Number of lymphocytes. (e) Neutrophil-lymphocyte ratio. (f) Monocyte-lymphocyte ratio.

The changes of total leukocyte and leukocyte populations were shown in Figure 1. Numbers of total leukocyte and neutrophils showed similar dynamic changes. Increases of them were observed as early as week 2 postinfection followed by a significantly transient decrease. Monkeys CM1936 and CM1938 showed a transient decrease and a continuous decrease in the number of monocytes during the infection period; however, CM1938 showed a slow time corresponding increase. Two monkeys (CM1936 and CM1938) exhibited a transient decrease in the number of lymphocytes after the infection, and a consistent decrease were observed in monkey CM1937. Neutrophil-lymphocyte ratio and monocyte-lymphocyte ratio were calculated from the numbers of neutrophils, monocytes, and lymphocytes, which highly resembled that of neutrophils and monocytes.

### 3.3. Peripheral T Lymphocyte Subsets

**Figure 2:**
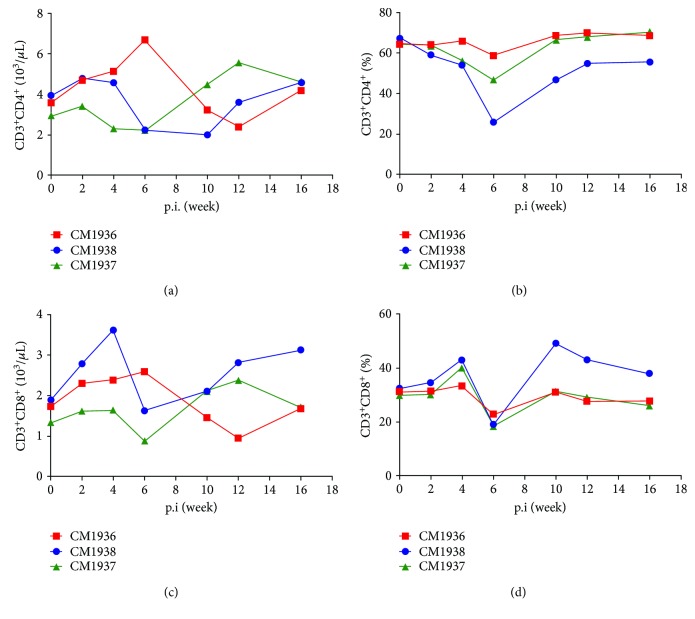
Dynamic changes of peripheral T lymphocyte subsets. (a) Numbers of CD3^+^CD4^+^ T cells. (b) The percentage of CD3^+^CD4^+^ T cell. (c) Numbers of CD3^+^CD8^+^ T cells. (d) The percentage of CD3^+^CD8^+^ T cell.

The dynamic changes of peripheral T lymphocyte subsets were similar for infected monkeys (Figure 2). All monkeys experienced moderate increases in numbers of peripheral CD3^+^CD4^+^ T cell and CD3^+^CD8^+^ T cell during the early infection course followed by a transient decrease. While the percentage of CD3^+^CD4^+^ T cell and CD3^+^CD8^+^ T cell showed a significant decrease at week 6 postinfection.

### 3.4. Cytokine Responses

**Figure 3:**
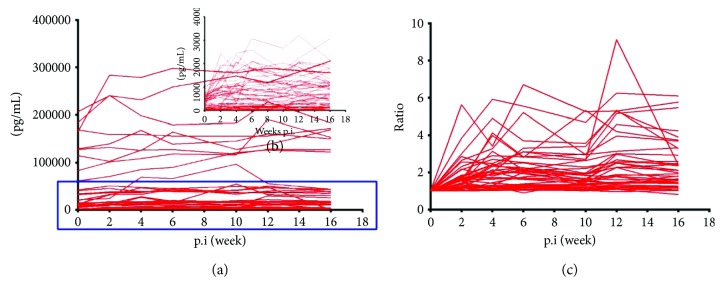
Dynamic changes of serum cytokines. (a) Showing the changes of all cytokines; (b) showing the changes of cytokines of low concentrations; (c) showing the ratios of serum concentrations postinfection by comparing the beginning values.

Dynamic changes of 44 cytokines were determined and analyzed. Serum concentrations were shown in Figures 3(a)-3(b). The increases of serum concentrations were observed for most cytokines as early as week 2 postinfection. And 36 cytokines presented increases for at least one time point. To analyze the characteristic of dynamic changes, the increase or decrease of serum concentrations postinfection was calculated by comparing the beginning values. The ratios were shown in Figure 3(c). The fluctuation of cytokine responses postinfection was further analyzed by comparing with the beginning values, and results of paired *t*-test were shown in Table 3. According to the characteristic of dynamic changes, these 44 cytokines were divided into 6 catalogs (Table 3). Cytokines of type 1 showed a sharp increase at early infection stage and kept high levels in most time of infection. Cytokines of type 2 showed a moderate increase at early infection stage followed by keeping at high levels. Type 3 presented a slight time corresponding increase during the infection period. Type 4 presented a transient increase during the infection period. For type 5, multipeak kinetics emerged during the infection period. There were no specific changes during the infection period in cytokines of type 6.

### 3.5. Antibody Responses

#### 3.5.1. Dynamic Changes of IgM

**Figure 4:**
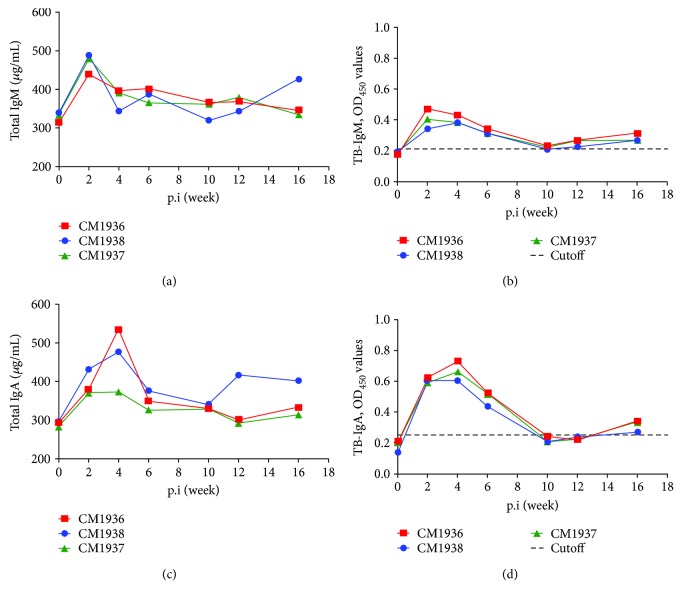
Dynamic changes of serum IgM and IgA. (a) Total IgM; (b) TB-IgM; (c) total IgA; (d) TB-IgA.

The serum concentrations of total IgM showed a significant increase at early infection stage and reached the high peaks at week 2 postinfection (Figure 4(a)). Then, the concentrations decreased slightly and returned to normal in animals CM1936 and CM1937. For animal CM1938, the next increase emerged from week 10 postinfection.

The dynamic changes of OD450 values of TB-IgM were similar to total IgM, showing a transient increase from weeks 2 to 10 postinfection (Figure 4(b)). Positivities of TB-IgM have been observed since week 2 postinfection for all animals, and negative reaction only emerged at week 10 postinfection in animal CM1938.

### 3.6. Dynamic Changes of IgA

Similar to IgM, the total IgA and TB-IgA also showed a transient increase at the early infection stage (Figures 4(c) and 4(d)), but the peaks emerged at week 4 postinfection for both total IgA and TB-IgA. The TB-IgA was negative for all animals from weeks 10 to 12 postinfection.

#### 3.6.1. Dynamic Changes of IgG

Dynamic IgG responses to PPD and 11 *M. tuberculosis* antigens were tested in sequential serum samples of infected monkeys by indirect ELISA.

**Figure 5:**
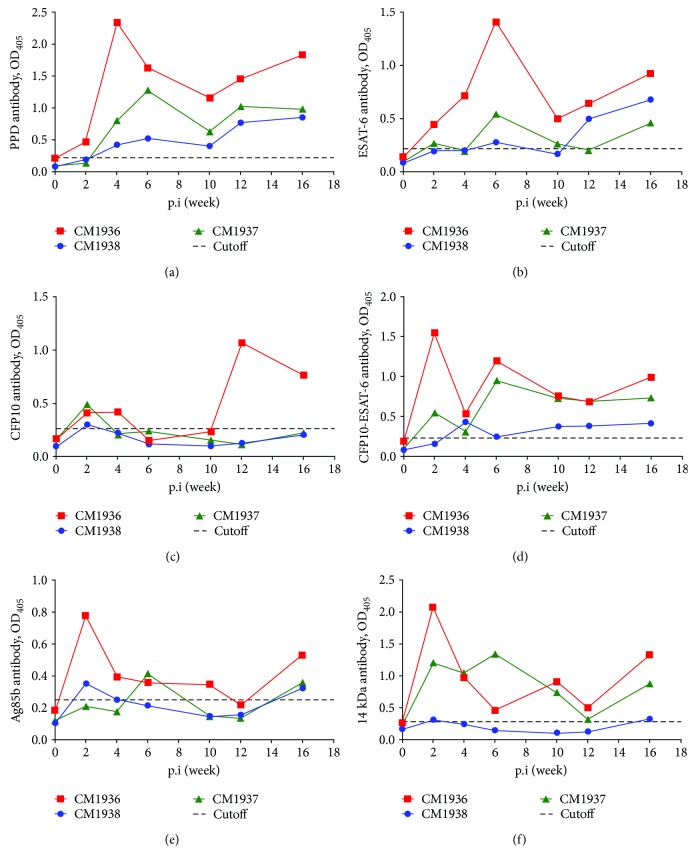
Positive IgG responses to PPD and 5 *M. tuberculosis* antigens. (a) PPD; (b) ESAT-6; (c) CFP10; (d) CFP10-ESAT-6; (e) Ag85b; (f) 14 kDa.

PPD, ESAT-6, CFP10, CFP10-ESAT-6, Ag85b, and 14 kDa antigens could be recognized by serum IgG of all animals from at least one time point during infection period (Figure 5). The time to IgG detection was as early as 2 weeks postinfection, which was defined as the earliest time point for a positive ELISA result. The IgG response characterizations of these antigens were similar to each other with a transient increase from 2 to 10 weeks postinfection, followed by a slow increase to terminal time point. Positive IgG against PPD and CFP10-ESAT-6 had been observed for all animals since week 2 postinfection. However, there was at least one time point for one or more animals showing negative antibody responses after 2 weeks postinfection for the remaining antigens.

**Figure 6:**
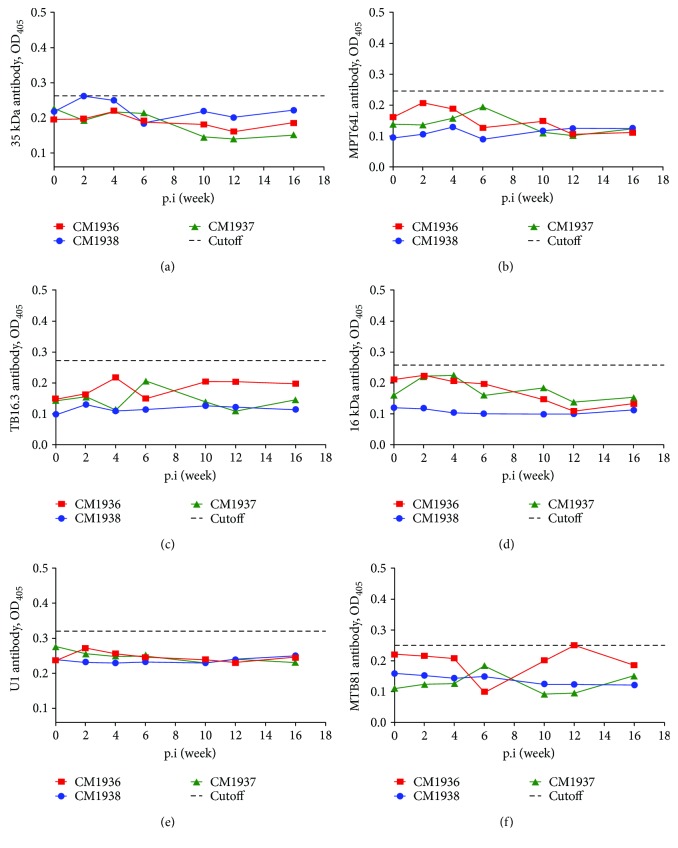
Negative IgG responses to 6 *M. tuberculosis* antigens. (a) 38 kDa; (b) MPT64L; (c) TB16.3; (d) 16 kDa; (e) U1; (f) MTB81.

Additionally, all animals presented negative IgG responses to 38 kDa, MPT64L, TB16.3, 16 kDa, U1, and MTB81 antigens during the infection period (Figure 6).

### 3.7. IgG Avidity

**Figure 7:**
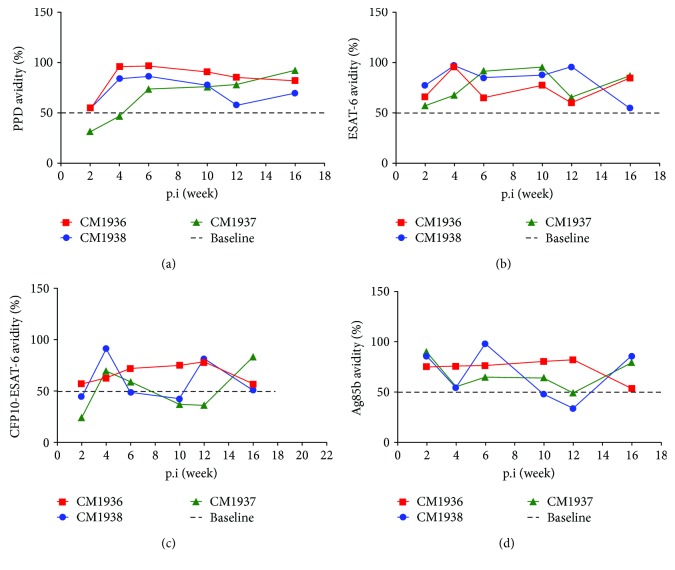
IgG avidities of antibodies against 4 *M. tuberculosis* antigens. (a) PPD; (b) ESAT-6; (c) CFP10-ESAT-6; (d) Ag85b.

In this study, we also detected the dynamic changes of IgG avidities to PPD, ESAT-6, CFP10-ESAT-6, and Ag85b (Figure 7). For seropositive responses to these antigens emerging as early as week 2 postinfection, IgG avidity was calculated from week 2 to week 16 postinfection. For most time points, the avidities were higher than 50%, which was considered as high-avidity antibody [17]. IgG avidity to CFP10-ESAT-6 in monkeys CM1937 and CM1938 showed a transient decrease after the first peak at week 4 postinfection.

## 4. Discussion

The incidence of pulmonary disease caused by NTM has been reported in the human population since the 1950s. *M. kansasii* and *M. intracellulare* are the most often emerging organisms. Similar to human patients, *M. kansasii* was one of the most responsible NTM species for NHP infection [7, 10–12]. However, research on NTM in NHP is limited.

In this study, we established an infectious NHP model by intratracheal inhalation of *M. kansasii* and performed clinical examinations on the animal model. During the infection period, positive TST reactions were observed in all 3 monkeys. Positive TB-IgM, TB-IgA, and IgG against PPD were observed as early as 2 weeks postinfection. At necropsy, monkey CM1938 presented consolidation on the surface and upon dissection of the left middle lung lobe, but no obvious lesions were observed in extrapulmonary organs of monkey CM1938 and all organs of the other 2 monkeys. *M. kansasii* was cultured from the left middle lung lobe of monkey CM1938. These infectious outcomes proved that all monkeys were successfully infected with *M. kansasii*. Monkeys CM1936 and CM1937 were more like a transient infection model of *M. kansasii*.

TST is the current mainstay of tuberculosis screening for NHP. However, previous reports also identified that natural *M. kansasii* infection could induce positive TST reactions in both rhesus macaques and squirrel monkeys [7, 10–12]. In our study, positive TST reactions were observed for all 3 monkeys during the infection period, which confirmed that *M. kansasii* infection could induce TST positivity. For the positive TST reactions only emerged from weeks 2 to 6 postinfection, results could be drawn that the PPD or mammalian old tuberculin-based TST was limited for diagnosing *M. kansasii* infection.

The clinical immune responses induced by the infection were analyzed by dynamic changes of total leukocyte and leukocyte populations, peripheral T lymphocyte subsets, and cytokine responses. The changes of total leukocyte, leukocyte populations, and peripheral T lymphocyte subsets were similar, showing a slight increase during the infection period. Multiplex cytokine assays identified that increases were observed in 36 out of 44 cytokines for all monkeys during the infection period. The ratios of increases by comparing the beginning values were moderate. No specific changes were found in 8 cytokines. Totally, the clinical immune responses were more moderate than those of experimental *M. tuberculosis* infected monkeys [13, 19].

For humoral immunity, IgM is the first antibody to be made by the immune system to fight a new infection. IgA can trigger antibody-dependent cell-mediated cytotoxicity (ADCC) by binding specifically to Fc*α*R. IgG is the most abundant type of antibody and protects against bacterial and viral infections. In this study, TB-IgM and TB-IgA were detectable as early as week 2 postinfection with a transient increase. And the peaks of serum concentrations of IgM were earlier than that of IgA and IgG.

For IgG detection, PPD was selected as candidate antigens because of its commercial maturation, robustness, and easy implementation. CFP10, ESAT-6, and CFP10-ESAT-6 were selected for their encoding CD-1 gene existing in both *M. tuberculosis* and *M. kansasii* [20]. Ag85b and 14 kDa antigens have high cross-reactivity with mycobacterial species [21]. The other 6 antigens (MPT64L, U1, 16 kDa, TB16.3, 38 kDa, and MTB81) can elicit strong antibody responses and are regarded as a surrogate of *M. tuberculosis* or *M. tuberculosis* complex [22, 23]. In the present study, positive IgG against PPD was observed as early as 2 weeks postinfection and kept at high levels after that. The antibody detection time was earlier than that of experimental *M. tuberculosis* infected monkeys [15]. PPD is mixed antigen of MTB and is also cross-reactive antigen to most mycobacteria. Our results also confirmed cross-reactive IgG responses to MTB-PPD for *M. kansasii* infection. ESAT-6 and CFP10 were encoded by RD1 (region of difference 1) gene that were deleted from most of mycobacteria. Besides MTB complex species, RD1 gene is also conserved in *M. kansasii*, though the gene in *M. kansasii* might reside in different segments [24, 25]. In this study, IgG against ESAT-6, CFP10, and CFP10-ESAT-6 in all animals reached positivities for at least on time point during the infection period. Ag85b is an abundantly secreted protein in *M. tuberculosis* culture that is not valuable in human TB diagnosis for its high cross-reactivity with mycobacterial species. Positive IgG response to Ag85b was also observed in *M. kansasii* infected monkeys. 14 kDa antigen is cross-reactive for mycobacterial species, and positive IgG responses also emerged in *M. kansasii* infected monkeys. However, IgG against 38 kDa, MPT64L, TB16.3, 16 kDa, U1, and MTB81 antigens never reached positivities.

For most time points, the IgG avidities were higher than 50%, which was considered as high-avidity antibodies. The results were different from viral infection in which high-avidity antibodies always emerged from a few weeks to months after primary infection. In our opinion, the following factors might affect the results. (a) Higher experimental infection dosage than natural infections might shorten the time course of primary infection. (b) Relative lower IgG responses might cause false high avidities.

In conclusion, we established a useful *M. kansasii* model in cynomolgus monkeys and used the model for immunity research. The identification of immune profiles of *M. kansasii* infection states suggests that *M. kansasii* is of weak virulence for cynomolgus monkeys and may induce cross-reactive antibody responses to *M. tuberculosis* antigens.

## Figures and Tables

**Table 1:** Information of selected cytokines.

Classification	Number	Cytokines
Panel 1	6	TNF-RII, IL19, CCL5/RANTES, MIF, MPO, Galectin-3bp
Panel 2	1	IL-23
Panel 3	37	IFN-gamma, TNF-alpha, TNF-RI, GM-CSF, IL-1 beta, IL-1ra, IL1-RII, IL2, IL2 R alpha, IL4, IL5, IL6, IL6R alpha, CXCL8/IL8, IL10, IL-15, IL17A, IL22, CCL2, CCL4, CCL7, CCL11, CCL13, CCL18, CCL22, CCL27, CXCL1/GRO alpha, CXCL4, CXCL10, CXCL12/SDF-1 alpha, CXCL13, CD14, CD27, CD30, CD40, NCAM-1/CD56, B7-H1/PD-L1/CD274

**Table 2:** The outcomes of monkeys infected with *M. kansasii*.

Infectious outcomes	Monkey 1936	Monkey 1937	Monkey 1938
*Clinical results*			
Symptoms and physical signs	Normal	Normal	Normal
Body weight	Normal	Normal	Normal
TST-positive reaction	Weeks 2~6 p.i.	Weeks 2~4 p.i.	Week 4 p.i.
Erythrocyte sedimentation rate	Normal	Normal	Normal
*Gross observation*			
Enlarged bronchial lymph nodes	N/A	N/A	N/A
Granuloma in lung	N/A	N/A	Lung consolidation
Granuloma in extrapulmonary organs	N/A	N/A	N/A
*Histopathology *			
Bronchial lymph nodes	Normal	Normal	Normal
Lung	Normal	Normal	Interstitial pneumonia
Extrapulmonary organs	Normal	Normal	Normal
*Bacterial load*			
Lung	N/A	N/A	5000 CFU/g^1^
Extrapulmonary organs	N/A	N/A	N/A

^1^The values were results of the left middle lobe.

**Table 3:** Results of comparisons with the beginning values and cytokine classification according to dynamic changes.

Type	Dynamic characteristic	Number	Cytokines	Compared to the beginning by paired *t*-test, falling within the range at week		
				*P* < 0.01	*P* < 0.05	*P* > 0.05
1	Sharp increase	7	TNF RII		2, 4, 10	6, 12, 16
			CXCL8/IL8		2, 4, 6, 10	12, 16
			IL10	6, 12, 16	2, 4	10
			CCL5/RANTES	16	2, 4, 10, 12	6
			CXCL4	6	2, 10, 12	4, 16
			CXCL10	6, 16	2, 4, 10, 12	
			CXCL13	2, 4, 6, 10, 16	12	

2	Moderate increase	8	IFN-gamma		2, 4, 12	6, 10, 16
			TNF-alpha		2, 4, 6, 10, 16	12
			GM-CSF		2, 4, 6, 10	12, 16
			IL-1 beta		4, 10, 12, 16	2, 6
			IL4		2, 6, 12, 16	4, 10
			IL17A		2, 4, 6	10, 12, 16
			CXCL12/SDF-1 alpha		12, 16	2, 4, 6, 10
			CD40	6, 10	4, 16	2, 12

3	Slight increase	8	TNF RI		2, 6, 16	4, 10, 12
			IL2	6		2, 4, 10, 12, 16
			IL15		2, 10	4, 6, 12, 16
			IL22			2, 4, 6, 10, 12, 16
			CCL2	4	10	2, 6, 12, 16
			CCL13		4, 6	2, 10, 12, 16
			CCL22	16	12	2, 4, 6, 10
			Galectin-3bp		4, 6, 12, 16	2, 10

4	A transient increase	7	IL6		2	4, 6, 10, 12, 16
			CCL18	2	4	6, 10, 12, 16
			CCL27			2, 4, 6, 10, 12, 16
			CXCL1/GRO alpha		4	2, 6, 10, 12, 16
			CD27		4, 10	2, 6, 12, 16
			B7-H1/PD-L1/CD274	4		2, 6, 10, 12, 16
			MIF		12	2, 4, 6, 10, 16

5	Multipeak kinetics	6	IL1 RA	6	16	2, 4, 10, 12
			IL1 RII		2	4, 6, 10, 12, 16
			IL23		12	2, 4, 6, 10, 16
			CCL4			2, 4, 6, 10, 12, 16
			CCL7		12	2, 4, 6, 10, 16
			CCL11	4, 12		2, 6, 10, 16

6	No specific changes	8	IL2 R alpha			2, 4, 6, 10, 12, 16
			IL5			2, 4, 6, 10, 12, 16
			IL6 R alpha			2, 4, 6, 10, 12, 16
			IL19			2, 4, 6, 10, 12, 16
			CD14			2, 4, 6, 10, 12, 16
			CD30			2, 4, 6, 10, 12, 16
			CD56			2, 4, 6, 10, 12, 16
			MPO			2, 4, 6, 10, 12, 16

## Data Availability

The data used to support the findings of this study are included within the article.
